# Nitroheterocyclic drug resistance mechanisms in *Trypanosoma brucei*

**DOI:** 10.1093/jac/dkv376

**Published:** 2015-11-17

**Authors:** Susan Wyllie, Bernardo J. Foth, Anna Kelner, Antoaneta Y. Sokolova, Matthew Berriman, Alan H. Fairlamb

**Affiliations:** 1 Division of Biological Chemistry and Drug Discovery, Wellcome Trust Building, College of Life Sciences, University of Dundee, Dundee DD1 5EH, Scotland, UK; 2 Wellcome Trust Sanger Institute, Hinxton, Cambridge CB10 1SA, UK

## Abstract

**Objectives:**

The objective of this study was to identify the mechanisms of resistance to nifurtimox and fexinidazole in African trypanosomes.

**Methods:**

Bloodstream-form *Trypanosoma brucei* were selected for resistance to nifurtimox and fexinidazole by stepwise exposure to increasing drug concentrations. Clones were subjected to WGS to identify putative resistance genes. Transgenic parasites modulating expression of genes of interest were generated and drug susceptibility phenotypes determined.

**Results:**

Nifurtimox-resistant (NfxR) and fexinidazole-resistant (FxR) parasites shared reciprocal cross-resistance suggestive of a common mechanism of action. Previously, a type I nitroreductase (NTR) has been implicated in nitro drug activation. WGS of resistant clones revealed that NfxR parasites had lost >100 kb from one copy of chromosome 7, rendering them hemizygous for *NTR* as well as over 30 other genes. FxR parasites retained both copies of *NTR*, but lost >70 kb downstream of one *NTR* allele, decreasing *NTR* transcription by half. A single knockout line of *NTR* displayed 1.6- and 1.9-fold resistance to nifurtimox and fexinidazole, respectively. Since NfxR and FxR parasites are ∼6- and 20-fold resistant to nifurtimox and fexinidazole, respectively, additional factors must be involved. Overexpression and knockout studies ruled out a role for a putative oxidoreductase (Tb927.7.7410) and a hypothetical gene (Tb927.1.1050), previously identified in a genome-scale RNAi screen.

**Conclusions:**

NTR was confirmed as a key resistance determinant, either by loss of one gene copy or loss of gene expression. Further work is required to identify which of the many dozens of SNPs identified in the drug-resistant cell lines contribute to the overall resistance phenotype.

## Introduction

There is an urgent need for new, safer and effective treatments for the diseases caused by the protozoan parasites *Trypanosoma brucei*, *Trypanosoma cruzi* and *Leishmania* spp. In the search for more effective drugs for these ‘neglected diseases’, researchers have chosen to reassess the therapeutic value of nitroaromatic compounds, previously avoided in drug discovery programmes due to perceived toxicity issues. This renewed interest largely stems from the success of nifurtimox/eflornithine combination therapy (NECT) for the treatment of the Gambian form of human African trypanosomiasis (HAT). Treatment with NECT, consisting of oral nifurtimox, a nitrofuran drug also used against Chagas' disease, combined with eflornithine infusions, has resulted in cure rates of ∼97% leading to its inclusion on the WHO Essential Medicines List.^1^ Since its introduction in 2009, NECT has rapidly become the treatment of choice for late-stage *T. brucei gambiense* HAT and is now being used to treat >60% of cases (http://www.doctorswithoutborders.org). In the wake of NECT, the Drugs for Neglected Diseases Initiative (DNDi) initiated a screen of previously abandoned nitroheterocyclics and rediscovered the 2-substituted 5-nitroimidazole fexinidazole from Hoechst (Hoe 239), first shown to have antitrypanosomal activity almost 30 years ago.^2^ In 2009, fexinidazole entered Phase I clinical trials and is currently undergoing Phase II/III assessment. As the first new clinical drug candidate for >30 years, there are now high hopes that this nitroimidazole can become the first orally available drug for both the haemolymphatic and meningoencephalitic stages of HAT. In addition, the DNDi is now undertaking a Phase II proof-of-concept study to evaluate fexinidazole for the treatment of primary visceral leishmaniasis in Sudan (www.dndi.org).^3^ Such is the reversal in their fortunes; there are now several nitro drugs, at various stages of development, populating the drug discovery pipelines of all three trypanosomatid diseases (www.dndi.org).^3,4^

Given the prominence of nitro drugs currently in clinical development, concerted efforts are now being made to elucidate their mechanisms of action and potential mechanisms of drug resistance. In African and South American trypanosomes, the mode of action of nifurtimox involves reductive activation via an NADH-dependent bacterial-like nitroreductase (NTR)^5–7^ with the formation of a cytotoxic, unsaturated open-chain nitrile derivative.^5^ Resistance to nifurtimox in laboratory-generated clones of *T. cruzi* is associated with loss of *NTR*.^8^ Moreover, nifurtimox-resistant (NfxR) *T. brucei* are cross-resistant to fexinidazole *in vitro* and *in vivo*.^9^ Collectively, these studies are suggestive of a common mechanism of action for these two nitroaromatic compounds with NTR pivotal to their bioactivation. This hypothesis was further substantiated in studies with *Leishmania donovani* where overexpression of a leishmanial homologue of NTR increased susceptibility to fexinidazole by 15-fold and nifurtimox by 19-fold.^3^

Reliance on a single enzyme, such as NTR, for drug activation has the potential to leave nitroaromatics vulnerable to the emergence of drug resistance. However, NTR activity has proven to be an absolute requirement for virulence in the trypanosomatids.^8,10,11^ In recent studies with *T. cruzi*, laboratory-generated benznidazole-resistant parasites were found to have lost a single copy of the NTR gene, with mutations arising in the remaining copy of the gene that abolished enzyme activity.^10^ The virulence of these ‘functional null’ parasites was so reduced that the capacity for such highly drug-resistant *T. cruzi* parasites to spread within the population would be severely compromised. Thus, the requirement to retain NTR activity may limit the maximum levels of nitro drug resistance achievable with NTR-activated drugs such as nifurtimox, benznidazole and fexinidazole. The potential for NTR-unrelated mechanisms of resistance to these drugs has rarely been considered. In order to investigate this possibility, here we use genome-wide sequencing to study the resistance mechanisms at play in bloodstream trypanosomes resistant to fexinidazole and nifurtimox.

## Materials and methods

### Cell lines and culture conditions

*T. brucei* bloodstream-form ‘single marker’ S427 (T7RPOL TETR NEO) and drug-resistant cell lines were cultured at 37°C in HMI9-T medium^12^ supplemented with 2.5 μg/mL G418 to maintain expression of T7 RNA polymerase and the tetracycline repressor protein. Cultures were initiated with 1 × 10^5^ cells/mL and subcultured when cell densities approached 1–2 × 10^6^ cells/mL.

In order to examine the effects of inhibitors on the growth of these parasites, triplicate cultures containing the inhibitor were seeded at 1 × 10^5^ trypanosomes/mL. Cell densities were determined after culture for 72 h as previously described.^13^ EC_50_ values were determined using the following two-parameter equation by non-linear regression using GraFit:

(1) $$y=\frac{100}{1+{\left([I]/\text{E}{\text{C}}_{50}\right)}^{m}}$$

where the experimental data were corrected for background cell density and expressed as a percentage of the uninhibited control cell density. In equation (1), [I] represents the inhibitor concentration and *m* is the slope factor.

### Drugs

Nifurtimox used in this study was a kind gift provided by Bayer, Argentina. Fexinidazole was synthesized in-house as previously described.^9^

### Generation of drug-resistant parasites

An NfxR line was generated by subculturing WT *T. brucei* in the continuous presence of nifurtimox.^9^ Starting at a sublethal concentration of 1.5 μM, the drug concentration in the culture media was increased in a stepwise manner, usually by 2-fold. After a total of 140 days in culture, when trypanosomes were able to survive and grow in 50 μM nifurtimox, the resulting *T. brucei* line (designated NfxR) was cloned by limiting dilution in the absence of nifurtimox. Three clones (NfxR1, NfxR2 and NfxR3) displaying the highest resistance to nifurtimox were selected for further studies. Resistance to fexinidazole was generated in *T. brucei* in a similar manner, starting at 1.0 μM fexinidazole. Fexinidazole-resistant (FxR) clones were derived from the resulting FxR line after 137 days in culture and clones FxR1, FxR2 and FxR3 were selected for further analysis.

### Generation of knockout and overexpression constructs

The primers used are summarized in Table S1 (available as Supplementary data at *JAC* Online) and were designed using the *T. brucei NTR* sequence in TriTrypDB (Tb427.07.7230) as a template. The accuracy of all assembled constructs was verified by sequencing. *NTR* gene replacement cassettes were generated by amplifying a region of DNA encompassing the 5′UTR, ORF and 3′UTR of *T. brucei* NTR from genomic DNA with primers 5′UTR-NotI_F and 3′UTR-NotI_R, using *Pfu* polymerase. An endogenous NotI site present within the 3′UTR was silenced using primers 3′UTR_mut_G89C_F and 3′UTR_mut_G89C_R and the QuikChange Lightning Site-Directed Mutagenesis Kit (Stratagene) as per the manufacturer's guidelines. This mutated sequence was then used as a template for the amplification of the individual regions used in the assembly of replacement cassettes containing the selectable drug resistance genes puromycin *N*-acetyl transferase (*PAC*) and hygromycin phosphotransferase (*HYG*) as previously described.^14^ The gene replacement construct (*PAC*) of the *T. brucei* gene Tb927.1.1050 was generated in an identical manner. The gene replacement construct (*HYG*) of the *T. brucei* gene Tb927.7.7410, encompassing the 450 bp upstream and downstream of the ORF, was commercially synthesized (GeneArt, Invitrogen).

To generate an overexpression construct of the *T. brucei* hypothetical gene annotated Tb927.7.7410, the ORF was amplified from genomic DNA using sense and antisense primers and cloned into pCR-Blunt II-TOPO (Invitrogen). The pCR-Blunt II-TOPO-Tb927.7.7410 construct was then digested with HindIII and PacI and the resulting fragment ligated into the HindIII/PacI cloning site of a modified pLew82 tetracycline-inducible expression vector,^14,15^ resulting in the introduction of a C-terminal triple haemagglutinin (HA) epitope tag into the expressed protein. Similarly, the *NTR* gene and a mutated version of the *NTR* gene, encoding a mutation of residue 106 from Val to Ile, was generated by site-directed mutagenesis with the NTR^MUT^ primers (Table S1) as described above. The mutated gene was then ligated into the HindIII/PacI cloning site of the original pLew82 tetracycline-inducible expression vector.^15^

### Generation of transgenic cell lines

Mid-log bloodstream trypanosomes were transfected with either knockout or recovery constructs using the Human T-Cell Nucleofector kit and nucleofector (Amaxa, program X-001). Following transfection, cells were allowed to grow for 16–24 h prior to appropriate drug selection (hygromycin and puromycin at 4 and 0.1 μg/mL, respectively). Cloned cell lines were generated by limiting dilution, maintained in selective medium and removed from drug selection for one passage prior to experiments.

### Verification of single gene knockouts (SKO) by PCR

PCR primer pairs 5′UTR_−595_F and 3′UTR_+600_R (outer primers) and 5′UTR_−107_F and 3′UTR_+152_R (inner primers) (Table S1) were used to verify the removal of a single allelic copy of *NTR* from bloodstream parasites. Genomic DNA from putative NTR^SKO^ cell lines was prepared and PCR was carried out using *Pfu* polymerase. Similar confirmatory studies were carried out with primer pairs 5′UTR_+500 and HYG R for Tb927.7.7410^SKO^ cell lines (Table S1).

### Southern blot analyses of transgenic T. brucei cell lines

The 5′UTR of *T. brucei* Tb927.1.1050 was amplified by PCR [using the primers previously described for the cloning of the single knockout (SKO) construct] and the PCR DIG Probe Synthesis Kit (Roche). The resulting DIG-labelled product was used as a probe. Samples of genomic DNA (5 μg) from WT and transgenic cell lines were digested with appropriate restriction endonucleases; then, the digestion products were separated on a 0.8% agarose gel and transferred onto a positively charged nylon membrane (Roche). The membrane was hybridized overnight in DIG Easy Hyb solution (Roche) at 42°C with the DIG-labelled 5′UTR probe (2 μL of PCR product). Following hybridization, membranes were washed twice in low-stringency conditions (25°C, 5 min, 1× SSC with 0.1% SDS) and twice in high-stringency conditions (68°C, 15 min, 0.5× SSC with 0.1% SDS), where 1× SSC comprises 150 mM sodium chloride and 50 mM sodium citrate (pH 7.0). Bound probe was detected using the DIG immunological detection kit (Roche) as per the manufacturer's instructions.

### Western blot analysis of T. brucei cell lysates

Overexpression of the protein encoded by the gene Tb927.7.7410 was induced by the addition of tetracycline (2 μg/mL) for 72 h prior to analysis by western blotting. *T. brucei* whole-cell extracts (1 × 10^7^ parasites per lane) were separated by SDS–PAGE and subsequently transferred onto nitrocellulose. After blocking with 5% skimmed milk in PBS for 1 h, blots were incubated with anti-HA mouse monoclonal antibody (Roche, 1 : 500), washed in PBS containing 0.1% (v/v) Tween 20 and then incubated with a secondary antibody (horseradish peroxidase-conjugated antimouse IgG, R&D Systems, Minneapolis, MN, USA; 1 : 5000 dilution). Immunoblots were developed using the ECL Plus (enhanced chemiluminescence) system from GE Healthcare (Piscataway, NJ, USA).

### Quantitative PCR (qPCR)

Levels of *NTR* transcript in resistant and WT parasites were analysed by qPCR using telomerase reverse transcriptase (*TERT*, Tb11.01.1950) as a reference gene. RNA was prepared from ∼1 × 10^7^ parasites using the RNeasy Kit (Qiagen) as per the manufacturer's instructions. cDNA was then generated from these samples using the iScript cDNA Synthesis Kit (Bio-Rad). All qPCR reactions were performed with PerfeCTa SYBR Green FastMix for iQ using the primer sets described in Table S1 and a Bio-Rad CFX96 Real-Time PCR Detection System. All statistical analyses are unpaired, two-tailed *t*-tests with a 95% CI and were performed using GraphPad Prism software.

### WGS and genomic variant analysis

Genomic DNA was prepared from *T. brucei* clones NR-B_427 (NfxR1), NR-D_427 (NfxR2), NR-G_427 (NfxR3), FR-A_427 (FxR1), FR-C_427 (FxR2) and FR-E_427 (FxR3). For each sample, 5 μg of genomic DNA was used to produce amplification-free Illumina libraries with fragment sizes of 350–450 bp.^16^ Sequencing was carried out on an Illumina HiSeq 2000 sequencer according to the manufacturer's standard sequencing protocol and yielded 33.6–43.8 million reads of 100 bp length per library for the drug-resistant lines. To allow direct comparison of genetic variant calls from these data with variant calls from shorter reads of an Illumina sequencing run of the *T. brucei* reference strain Lister 427 (lane 5189_8, 67.8 million reads of 76 bp length), the 100 bp reads were hard clipped to a length of 76 bp. The resulting datasets represented a nominal sequencing coverage of the 35 Mb *T. brucei* genome of ∼73- to 95-fold (drug-resistant strains) and 147.2-fold (Lister 427). The Illumina data were aligned against the *T. b. brucei* TREU927 reference genome assembly^17^ using SMALT v0.7.4 (http://www.sanger.ac.uk/resources/software/smalt/). For variant calling, the alignment was run employing an exhaustive search (−x) and with parameters wordlen = 13 (−k), skipstep = 1 (−s), minscor = 0.65 (−m) and insertmax = 1000 (−i). To assess relative read coverage and copy number variations, the alignment runs were repeated using the same parameters but additionally with repetitive mapping (−r) enabled. Variants were called using SAMtools v0.1.19 mpileup (−Q 15 for baseQ/BAQ filtering) and BCFtools.^18^ To exclude the hypervariable subtelomeric regions, only variants found in the following chromosomal core regions were included in the downstream analyses: Tb927_01_v4: 202 695–988 120; Tb927_02_v4: 259 723–1 161 408; Tb927_03_v4: 146 614–1 602 829; Tb927_04_v4: 80 380–1 467 268; Tb927_05_v4: 72 088–1 366 595; Tb927_06_v4: 111 409–1 414 033; Tb927_07_v4: 26 571–2 177 541; Tb927_08_v4: 135 192–2 476 033; Tb927_09_v4: 325 850–2 394 987; Tb927_10_v5: 55 698–3 993 940; and Tb927_11_01_v4: 36 585–4 482 610. SNP calls were further filtered for all of the following: for a minimum of eight ‘high-quality’ base calls (‘DP4’); for a minimum phred-scale QUAL score of 20; for a maximum phred-scale likelihood of the best genotype call of 5 (‘PL1’); for a minimum phred-scale likelihood of the second best genotype call of 10 (‘PL2’); for a minimum strand bias *P* value of 0.01 (first of ‘PV4’); for a maximum ratio of conflicting base calls for homozygous genotypes of 5% and, for positions with a minimum and maximum read depth of three times the median read depth observed for that chromosome: for a minimum mapping quality of 20 and for a minimum distance of 10 nucleotides from the nearest INDEL call. Files in Variant Call Format (VCF) listing all 190 064 genomic positions at which any one of the six drug-resistant and the parental parasite lines had a variant call are available at http://dx.doi.org/10.15132/10000107. The raw sequence data are available under the following accession numbers at the European Nucleotide Archive (http://www.ebi.ac.uk/ena): Tb427 WT (SM_S427): ERS012470; NR-B_427: ERS017528; NR-D_427: ERS017529; NR-G_427: ERS017530; FR-A_427: ERS017531; FR-C_427: ERS017532; and FR-E_427: ERS017533.

## Results

### NfxR and FxR trypanosomes

In our previous studies we investigated the ease with which resistance to nifurtimox and fexinidazole could be generated *in vitro* in *T. brucei* (S427) bloodstream trypanosomes.^9^ In our current study, resistance was readily achievable with the resulting NfxR (NfxR1–2) and FxR (FxR1–2) clones being ∼6- and ∼11-fold resistant to nifurtimox and fexinidazole, respectively, compared with WT (Table 1). NfxR trypanosomes demonstrated cross-resistance *in vitro* and *in vivo* to fexinidazole, with FxR parasites similarly cross-resistant (Table 1). The EC_50_ values determined for these cell lines in our current study are in good agreement with previously published data.^9^ However, in this study, the Hill slope values for the FxR cell lines (1.2–1.5) are half as steep in comparison with the WT parasite (2.6) and its genetically manipulated derivatives. This feature was not evident in our previous study (e.g. Hill slopes of 1.6 versus 1.4 for WT and FxR parasites, respectively) and is unique to FxR parasites. The significance of this observation is not clear, but may suggest some differences in the mechanisms of action of fexinidazole and nifurtimox. The reciprocal cross-resistance relationship observed here with NfxR and FxR parasites strongly supports our hypothesis that a predominantly similar mechanism of action is involved for these two compounds.^9^ However, it is notable that the level of resistance to fexinidazole in the selected lines is always greater than the resistance to nifurtimox, regardless of which drug was used in the selection. Here, the resistance mechanisms exploited by both resistant lines are studied.

**Table 1.** Susceptibility of WT, nitro drug-resistant and transgenic *T. brucei* cell lines to nifurtimox and fexinidazole

Cell line	Nifurtimox, EC_50_, μM^a^	Resistance factor	Fexinidazole, EC_50_, μM^a^	Resistance factor
WT	3.4 ± 0.09 (2.7)	1	1.4 ± 0.13 (2.6)	1
NfxR				
clone 1	19.7 ± 1.4 (2.1)^b^	5.8	14.5 ± 1.5 (1.2)^b^	10.4
clone 2	22.3 ± 1.6 (2.2)^b^	6.5	16.8 ± 2.4 (1.4)^b^	12
FxR				
clone 1	19.1 ± 1.0 (2.4)^b^	5.6	13.2 ± 1.0 (1.5)^b^	9.4
clone 2	18.3 ± 0.9 (3.4)^b^	5.4	13.0 ± 1.1 (1.2)^b^	9.3
NTR^SKO^				
HYG	5.8 ± 0.1 (2.3)^b^	1.7	2.6 ± 0.2 (2.2)^c^	1.9
PAC	6.5 ± 0.1 (1.9)^b^	1.9	2.8 ± 0.1 (2.1)^c^	2.0
Tb927.1.1050^SKO^				
clone 1	2.2 ± 1.0 (2.2)^d^	1.0	1.9 ± 0.7 (2.8)	1.4
NTR^SKO^/Tb927.1.1050^SKO^				
clone 1	3.8 ± 0.2 (2.4)^d^	1.8	2.8 ± 0.2 (2.5)	2.0
clone 2	4.0 ± 0.3 (2.0)^d^	1.7	2.7 ± 0.4 (2.5)	1.9
Tb927.7.7410^OE^				
clone 1	3.1 ± 0.2 (3.4)	0.9	1.2 ± 0.05 (2.7)	0.9
clone 2	3.1 ± 0.2 (2.3)	0.9	1.3 ± 0.06 (2.1)	0.9
TbNTR_V106I_^OE^				
clone 1	0.38 ± 0.01 (2.1)^b^	0.11	ND^e^	—
TbNTR^OE^				
clone 1	0.32 ± 0.01 (2.0)^b^	0.09	ND^e^	—

^a^Results are the mean and standard error of at least three independent experiments. Slope factors are indicated in brackets.

^b^Unpaired *t*-test determined these EC_50_ values to be significantly different from WT values with a statistical significance of *P* < 0.0001.

^c^Unpaired *t*-test determined EC_50_ values to be significantly different from WT values with a statistical significance of *P* ≤ 0.001.

^d^The EC_50_ value for nifurtimox against WT parasites in this dataset was 2.3 ± 0.3 μM with the resistance factors calculated accordingly.

^e^Not determined.

### Role of NTR in NfxR and FxR drug resistance

Bioactivation of nifurtimox and fexinidazole is catalysed by type I NTR enzymes in both *T. brucei* and *Leishmania*.^3,7^ The potential role of this enzyme in the nitro drug resistance mechanisms of NfxR and FxR trypanosomes was investigated. To determine whether any mutations within the *NTR* genes of these cell lines could account for nitro drug resistance, the ORF of TbNTR, flanked by 100 bp at the 5′-terminus and 150 bp at the 3′-terminus, was amplified by PCR from the genomic DNA of each cell line and analysed by in-house sequencing in Dundee. The *NTR* gene in all NfxR clones was found to contain a point mutation that encoded a single amino acid change (V106I). This mutation was not present in any of the FxR clones. To assess the potential impact of this amino acid change on the activity of NTR, a tetracycline-inducible ectopic copy of the mutant gene was introduced into WT cells. All tetracycline-induced clones, derived from the resulting TbNTR_V106I_-overexpressing *T. brucei* cell line, were found to be ∼9-fold more susceptible to nifurtimox than the parental WT cells with a mean EC_50_ value 0.38 ± 0.01 μM (Table 1). This is very similar to the 10.6-fold increase in susceptibility seen with cells overexpressing WT NTR (EC_50_ value 0.32 ± 0.01 μM). Collectively, these data indicate that mutant TbNTR_V106I_ is capable of activating nifurtimox in bloodstream trypanosomes and that this mutation is unlikely to play any significant role in nitro drug resistance.

**Figure 1.**
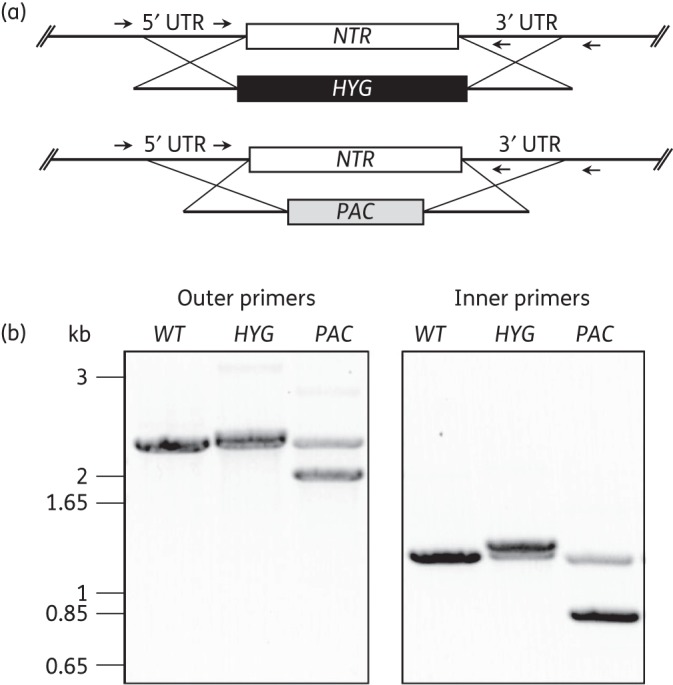
Generation and analysis of *NTR* single gene knockout cell lines. (a) A schematic representation of *NTR* gene deletion in the knockout *T. brucei* cell lines SKO*^HYG^* (top) and SKO*^PAC^* (bottom). Arrows indicate the position of PCR primer pairs 5′UTR_−595_F and 3′UTR_+600_R (outer pair) and 5′UTR_−107_F and 3′UTR_+152_R (inner pair). (b) Agarose gel electrophoresis of PCR products generated with the primer pairs indicated in (a). The template genomic DNA was derived from WT *T. brucei*, SKO*^HYG^* clone 1 and SKO*^PAC^* clone 1.

An initial Southern analysis of genomic DNA from the nitro drug-resistant trypanosomes appeared to suggest that a single allele of *NTR* has been lost in both NfxR1 and NfxR2 cell lines. However, FxR clones appeared to maintain both *NTR* alleles (Figure S1). The impact of a reduction in the *NTR* copy number on nitro drug susceptibility was investigated by generating *NTR* SKO cell lines. A single copy of the *NTR* gene was replaced with either the hygromycin resistance gene (*HYG*) or the puromycin resistance gene (*PAC*) by homologous recombination and drug selection. The successful removal of single copies of the *NTR* gene from these diploid parasites was confirmed by PCR (Figure 1). The resulting SKO*^HYG^* and SKO*^PAC^* cell lines showed no major growth phenotype with doubling times of ∼8 h, similar to the 7.3 h doubling time of WT parasites. However, SKO*^HYG^* parasites were found to be 1.7- and 1.9-fold less susceptible than WT *T. brucei* to nifurtimox and fexinidazole, respectively (Table 1). Similarly, SKO*^PAC^* cells were 1.9-fold less susceptible to nifurtimox. Given that NfxR cells demonstrate a ∼6-fold shift in their susceptibility to nifurtimox, it is clear from these data that the loss of a single allele of *NTR* may not be entirely responsible for the levels of nitro drug resistance seen with these cells and additional factors may be involved.

### WGS of NfxR and FxR cell lines reveals large-scale losses of single alleles and numerous genetic variants

**Figure 2.**
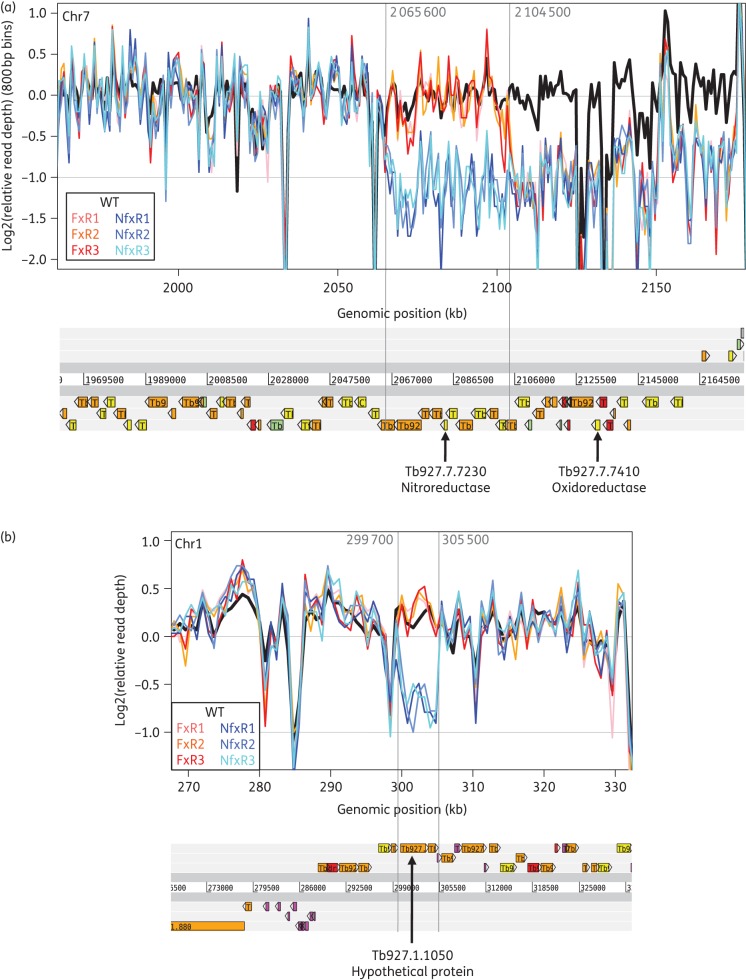
Hemizygous regions in the drug-resistant parasite lines identified by WGS. The read coverage plots show the relative read depth (analysed in windows of 800 bp width) by plotting the log2-based ratio of the read depth observed in a given window over the average read depth of the corresponding chromosome. A log2-based relative read depth of −1 therefore indicates a read coverage that is 0.5-fold that of the average read depth, which may be due to the loss of one of the two alleles in that region (hemizygosity). Note the variable but highly reproducible (across the different parasite lines) nature of the read coverage, which is caused by differences in sequence read mapping efficiency across the genome especially in regions of repetitive sequence. The location of genes along the chromosomes is indicated below the graphs. (a) The plot shows the 3′-end (the ‘right-hand side’) of the chromosome core region of chromosome 7. A 50% reduction in read depth is apparent for the three replicate NfxR lines for more than 100 kb starting from around genomic position 2 065 600 and similarly for the three replicate FxR lines starting from around genomic position 2 104 500. The genomic variants listed in Table S3 confirm the expected concomitant loss of heterozygosity in this region. The hemizygous region observed in the NfxR lines includes NTR, whereas the hemizygous region in the FxR lines does not, although it still apparently overlaps the polycistronic transcription unit in this region. (b) A 5.8 kb hemizygous region on chromosome 1 observed only in the three replicate NfxR lines.

In an attempt to identify any additional factors that may be involved in the drug resistance phenotypes of NfxR and FxR cell lines, the genomes of each clone were sequenced. The sequence data readily confirmed the loss of one copy of the NTR locus for the NfxR parasites, rendering a region of >100 kb hemizygous (Figure 2a). This includes 32 putative genes, including a putative oxidoreductase (Tb927.7.7410) (Table S2). For the FxR parasites, the 3′-end of the chromosomal core region of chromosome 7 was likewise found to be present in only a single copy, but here the hemizygous region started ∼20 kb downstream of the NTR locus (Figure 2a) with hemizygosity for 22 genes (Table S2). In addition, the sequencing data revealed another 5.8 kb region on chromosome 1, encompassing Tb927.1.1050, that has been lost solely in the NfxR cell lines (Figure 2b). Comparing the genotype calls of the drug-resistant lines with those of the parental strain also uncovered many dozens of SNPs that are predicted to result in non-synonymous amino acid changes that could potentially contribute to the observed drug resistance phenotypes (Table S3). Unfortunately, because of the repetitive nature of the *T. brucei* genome, which contains many duplicated genes, such lists of genomic variant candidates are often too long to be experimentally verified one by one. Below, we report the experimental follow-up of the most prominent genetic variants.

### Does the putative oxidoreductase (Tb927.7.7410) play a role in nitro drug resistance?

**Figure 3.**
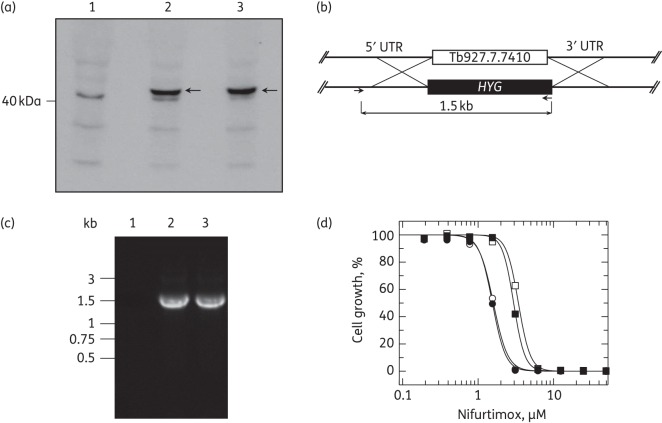
Overexpression and single gene knockout of a putative oxidoreductase encoded by Tb927.7.7410 in bloodstream trypanosomes. (a) Overexpression of the HA-tagged protein encoded by Tb927.7.7410 was induced by the addition of tetracycline 72 h prior to analysis. Cell lysates (1 × 10^7^ parasites in each lane) were separated by SDS–PAGE and analysed by western blotting with a rabbit anti-HA antibody. Lysates were derived from WT cells (lane 1) and cells transfected with pLEW82-7410-HA_3_ (clone 1 in lane 2 and clone 2 in lane 3). Arrows indicate the position of Tb927.7.7410. (b) Schematic representation of the ORF of Tb927.7.7410 flanked by 5′UTR and 3′UTR regions. Successfully replacing a single copy of this gene with *HYG* allows a PCR fragment of 1.5 kb to be generated by specific primers designed to the 5′UTR (outside of the gene replacement construct) and *HYG*. (c) Agarose gel electrophoresis of PCR products generated with the primer pairs indicated in (b). The template genomic DNA was derived from WT *T. brucei* (lane 1), Tb927.7.7410^SKO^ clone 1 (lane 2) and *NTR*/Tb927.7.7410^SKO^ (lane 3). (d) EC_50_ values of 1.6 ± 0.03, 1.5 ± 0.03, 3.4 ± 0.06 and 3.1 ± 0.04 μM were determined for nifurtimox against WT (open circles), Tb927.7.7410^SKO^ (filled circles), *NTR*^SKO^ (open squares) and Tb927.7.7410/*NTR*^SKO^ (filled squares) trypanosomes, respectively.

Loss of genetic material from a single copy of chromosome 7 in NfxR and FxR parasites resulted in the deletion of a putative oxidoreductase. Since this class of enzymes has the potential to catalyse the reduction of nitro drugs, the enzyme's role in the activation of fexinidazole and nifurtimox was investigated. A triple HA epitope-tagged version of the putative oxidoreductase, encoded by gene Tb927.7.7410, was overexpressed in bloodstream trypanosomes. Expression of the putative oxidoreductase in these transgenic parasites was confirmed by western blotting using an anti-HA mouse monoclonal antibody (Figure 3a). An extra band at 42 kDa is visible in the overexpressing lines consistent with the predicted molecular mass of the tagged protein. Two clonal cell lines expressing the tagged oxidoreductase were then assessed for their susceptibilities to both nifurtimox and fexinidazole; these clones were found to be equally susceptible to the nitroaromatic compounds as WT parasites (Table 1). A proteomic study of the mitochondria of procyclic *T. brucei*^19^ established that this oxidoreductase may be associated with subcomplex Iα of the respiratory chain. However, preliminary immunofluorescence studies suggested that the HA-tagged enzyme was predominately localized to the cytosol of bloodstream trypanosomes. Since we cannot confirm whether or not our overexpressed, tagged oxidoreductase is correctly localized in the cell, or indeed functional, additional studies were carried out. A single copy of Tb927.7.7410 was replaced in WT trypanosomes (Tb927.7.7410^SKO^) and also in *NTR*^SKO^ (*NTR*/Tb927.7.7410^SKO^) parasites, thus mimicking the genetic composition of NfxR and FxR parasites (Figure 3b and c). Removal of a single copy of this gene was found to have no impact on the nitro drug susceptibility of either WT parasites or in cells lacking a single copy of *NTR* (Figure 3d)*.* Collectively, these data do not support a role for this putative oxidoreductase in the mechanism of action of, or resistance to, fexinidazole and nifurtimox in *T. brucei*.

### Hypothetical protein Tb927.1.1050

**Figure 4.**
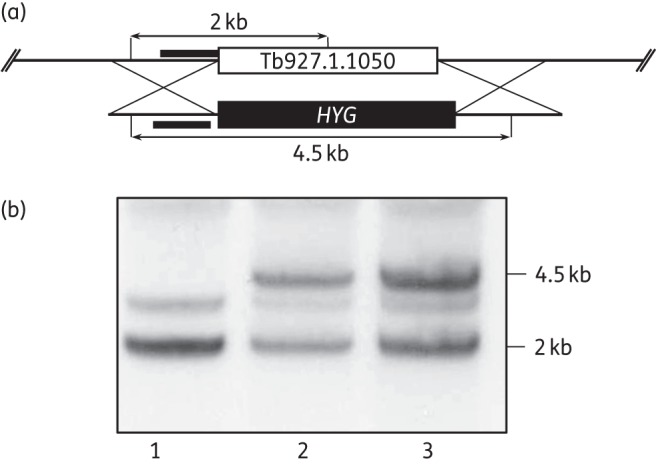
Generation of a Tb927.1.1050 single gene knockout in NTR SKO*^HYG^* cells. (a) The black bars represent the 5′UTR region upstream of the ORF of Tb927.1.1050 used as a probe in Southern blot analysis. SalI sites with expected fragment sizes are shown. The endogenous Tb927.1.1050 gene contains a SalI site, which results in a 2 kb band. Successful replacement of one allelic copy of the endogenous gene with *HYG*, which does not contain a SalI site, results in a 4.5 kb fragment. (b) Southern blot analysis of SalI-digested genomic DNA (∼5 μg) from *T. brucei* *NTR* SKO*^PAC^* cells (lane 1), *NTR* SKO*^PAC^* background and Tb927.1.1050 SKO*^HYG^* clone 1 cells (lane 2) and *NTR* SKO*^PAC^* background and Tb927.1.1050 SKO*^HYG^* clone 2 cells (lane 3). The 5′UTR of the gene Tb927.1.1050 was used as a probe. A faint non-specific band can be seen at ∼3 kb in all three lanes.

Genome-wide sequencing also revealed that one allelic copy of the gene Tb927.1.1050, which encodes a putative metallo-dependent phosphatase, was lost from the NfxR cloned cell lines. Changes to this uncharacterized gene drew our attention since it had previously been identified in genome-scale RNA interference target sequencing (RIT-seq) screens of nifurtimox in *T. brucei*.^20^ To investigate any potential role in nifurtimox resistance, a single copy of Tb927.1.1050 was replaced in WT trypanosomes (Tb927.1.1050^SKO^) and also in *NTR*^SKO^ parasites (Figure 4). Removal of a single copy of this gene was found to have absolutely no impact on the nitro drug susceptibility of either WT parasites or in cells lacking a single copy of *NTR* (Table 1). It is perplexing that this gene has been implicated in nitro drug resistance in both RIT-seq and WGS studies; however, our data do not support a role for this putative metallo-dependent phosphatase in the mechanism of action of, or resistance to, nifurtimox in *T. brucei*.

### NTR transcript levels in NfxR and FxR cell lines

Confirming our preliminary Southern analysis (Figure S1), genome-wide sequencing revealed that both NfxR clones had lost a single copy of *NTR* while FxR clones maintained both copies. However, a large section of chromosome 7 downstream of *NTR* appeared to have been deleted from single alleles of both the NfxR and FxR cell lines. This includes a divergent strand-switch region, i.e. a probable transcription start site for RNA polymerase II,^21^ between Tb927.7.7480 (a putative *trans*-sialidase) and Tb927.7.7490 (a conserved hypothetical protein). The impact of this deletion on the transcript levels of *NTR* was determined by qRT-PCR. Relative levels of *NTR* cDNA in WT, NfxR1 and FxR1 cell lines were normalized using the reference housekeeping gene telomerase reverse transcriptase (*TERT*). *NTR* transcript levels in NfxR1 and FxR1 clones were reduced to 0.42 ± 0.06 and 0.6 ± 0.07 of the levels seen in the WT parasites, respectively. The ∼50% reduction in the levels of *NTR* mRNA is consistent with the loss of a single allele of *NTR* from NfxR cells, already demonstrated by genome sequencing. Further, it suggests that the loss of genetic material downstream of *NTR* leaves FxR cells unable to transcribe one allele of *NTR* and that these cells are functionally hemizygous for NTR despite maintaining both allelic copies of this gene.

## Discussion

The pivotal role of type I NTR enzymes in the activation of nifurtimox, benznidazole and fexinidazole in trypanosomatids is well established.^3,7,8^ Therefore, it is no surprise that in our current study we confirm that reduced levels of *NTR* expression are principally responsible for resistance in our NfxR and FxR cell lines. Generation of resistance to nifurtimox in bloodstream trypanosomes resulted in the loss of over 100 kb from one allele of chromosome 7. This deletion rendered NfxR parasites hemizygous for *NTR* as well as over 30 other genes. FxR parasites retained both copies of *NTR*, but it appears that a deletion 20 kb downstream left these parasites unable to transcribe one allele of this gene. To our knowledge, this mechanism has not been observed previously in trypanosomes and underlines the importance of not only determining gene copy number, but also possible changes in the relevant transcription unit. Significant chromosomal deletions of this nature are a common phenomenon in trypanosomes,^10,22,23^ which are renowned for their genome plasticity.^24^

To quantify the impact of the loss of one *NTR* copy, an SKO cell line was generated. *NTR*^SKO^ trypanosomes demonstrated 1.6- and 1.9-fold resistance to nifurtimox and fexinidazole, respectively, compared with the parental WT line. Bearing in mind the levels of nitro drug resistance seen with our NfxR and FxR cell lines, these findings clearly indicate that other mechanisms, in addition to loss of expression of *NTR*, must contribute to resistance. Detection of a mutation within the remaining copy of *NTR* in NfxR parasites (NTR_V106I_) was eliminated as a resistance factor, because the resulting mutant protein retained its NTR activity. In our hands, it has proved impossible to obtain pure recombinant *T. brucei* NTR,^25^ unlike other reports on *T. cruzi* NTR,^8,10^ making it impossible to carry out more detailed enzymatic studies.

**Figure 5.**
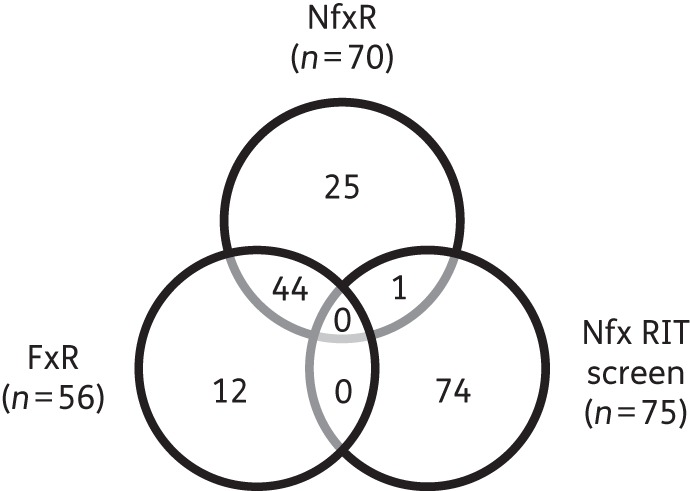
Venn diagram of overlapping gene ‘hits’ from RIT-seq screening of nifurtimox in *T. brucei* and of genes with high-confidence SNPs from WGS of NfxR and FxR trypanosomes. See Table S4 for further details.

In addition, we were able to effectively rule out a putative oxidoreductase, lost in the deletion of chromosome 7, as playing any role in resistance. In ruling out these factors, we must consider that 1 of the 44 SNPs common to both drug-resistant cell lines may be contributing to the drug resistance phenotype (Figure 5 and Table S4). The mechanisms that may lead to drug resistance in trypanosomatids can be generally classified into three categories: mechanisms that lower the drug levels within the cell; mechanisms that affect the main enzymes interacting with the drug (in the case of pro-drugs these can include the main activating proteins); and general defence and repair mechanisms.^26^ With this in mind, it is entirely plausible that a mutation within a specific target may reduce its affinity for the cytotoxic open-chain nitrile metabolite generated following bioreduction of nifurtimox.^7^ Alternatively, SNPs within transporters could reduce the uptake or increase the efflux of both nifurtimox and fexinidazole. A non-synonymous SNP was observed in a putative membrane transporter (Tb9.211.2900) in both NfxR and FxR clones (Table S4). Unfortunately, due to the sheer number of SNPs identified in the genomes of FxR and NfxR parasites, we are unable to systematically assess their roles in drug resistance.

The mechanisms underlying resistance to nifurtimox in *T. brucei* have also recently been assessed using a genome-scale RIT-seq screen.^20^ In this elegant study, a total of 75 genes were identified as being associated with nifurtimox resistance, with 8 genes found to have a strong association. Of these eight genes, six were found to directly or indirectly reaffirm the dominant role of the NTR in activation of nifurtimox. The major primary signature in the nifurtimox RIT-seq profile identified *NTR*. In addition, a putative flavokinase, believed to be involved in the conversion of riboflavin to FMN, the essential cofactor of NTR, was also identified. The remaining four signatures were assigned to genes involved in ubiquinone biosynthesis, supporting the hypothesis that NTR functions as an NADH dehydrogenase transferring electrons from NADH to ubiquinone generating ubiquinol.^8^ A direct comparison of the ‘hits’ from RIT-seq screening of nifurtimox and the SNPs identified in the genome sequence from NfxR and FxR cell lines confirmed that *NTR* was common to both the RIT-seq and NfxR datasets (Figure 5 and Table S4). In addition, a hypothetical gene (Tb927.1.1050), identified as a copy number variant in our NfxR dataset, was also identified via RIT-seq; however, our subsequent analysis could not demonstrate a role in nitro drug resistance. Collectively, these and other data continue to place trypanosomatid NTR enzymes firmly at the centre of the mechanisms of action/resistance of both nifurtimox and fexinidazole. It is surprising that there is so little overlap between the results of RIT-seq screening with nifurtimox and WGS of laboratory-generated NfxR cell lines. It is clear that both approaches have strengths and weaknesses. RNAi methodologies such as RIT-seq are unable to detect mutations that retain biological function whilst losing susceptibility to a specific inhibitor (e.g. SNPs in Figure 5). RIT-seq cannot detect gene amplifications or changes in regulatory elements that lead to resistance. Resistance involving two or more genes acting in concert cannot be detected using RIT-seq. The read density cut-off in RIT-seq ascribed as significant is somewhat arbitrary (>100) and may be set too high (e.g. Tb927.1.1050 is in the secondary read density category).^20^ Finally, short-term knockdown of essential pathways such as riboflavin or ubiquinone biosynthesis may have a long-term fitness cost in the drug selection approach. Moreover, the time taken to generate resistance to compounds in the laboratory combined with the intrinsically high mutation rate of trypanosomatids can make the identification of resistance-relevant SNPs extremely difficult. Indeed, the chemical stress that results from prolonged drug exposure can itself lead to mutations unrelated to the general mechanism of drug resistance. These studies illustrate the value of pooling datasets from different approaches in studies of drug resistance/mechanism of action.

The launch of NECT in 2009 demonstrated that nitro drugs could be used to treat trypanosomatid diseases safely and effectively. As a result, there has been a marked increase in the number of nitroaromatic compounds in development for these diseases,^27^ including for the first time a clinical candidate for visceral leishmaniasis.^3^ With the likelihood that this class of compounds will be more extensively used in the clinic, it is imperative that we understand not only the mechanisms of action of these drugs but also the likely mechanisms of drug resistance. In this and our previous studies, we have shown that NfxR *T. brucei* are cross-resistant to fexinidazole.^9^ It is of particular concern that our NfxR parasites were in fact considerably more resistant to fexinidazole than nifurtimox. Should this observation be repeated in a clinical setting, widespread resistance to nifurtimox as part of NECT could compromise the future use of fexinidazole. In this case, cross-resistance is largely due to their common NTR-mediated mode of action. This illustrates the risk of simultaneously developing multiple nitroaromatic drugs, with potentially similar mechanisms of action, for the treatment of trypanosomatid diseases. In such a scenario, a single drug resistance mechanism could result in multiple compound failures. Thus, as novel nitro drugs such as DNDI-VL-2098 are developed,^4^ defining their mechanisms of action should be a matter of urgency.

## Funding

This work was supported by grants from the Wellcome Trust (079838 and 100476, to A. H. F., and 098051 to the Wellcome Trust Sanger Institute) and a studentship from the Biotechnology and Biological Sciences Research Council (BBSRC) to A. Y. S.

## Transparency declarations

None to declare.

## Supplementary data

Figure S1 and Tables S1 to S4 are available as Supplementary data at *JAC* Online (http://jac.oxfordjournals.org/).

Supplementary Data
