# Supplementary material for: Nitroheterocyclic drug resistance mechanisms in Trypanosoma brucei
Source: J Antimicrob Chemother. 2015 Nov 17;71(3):625–34. doi: 10.1093/jac/dkv376 (PMC4743696; doi:10.1093/jac/dkv376)
Supplement: Supplementary Data [file supp_71_3_625__index.html]

Nitroheterocyclic drug resistance mechanisms in Trypanosoma brucei — Nitroheterocyclic drug resistance mechanisms in Trypanosoma brucei — Supplementary Data 

# Nitroheterocyclic drug resistance mechanisms in *Trypanosoma brucei*

## Supplementary Data

Supplementary Data

- Supplementary Data1 - docx file
- Supplementary Data2 - xlsx file
