# Supplementary material for: Nitroheterocyclic drug resistance mechanisms in Trypanosoma brucei
Source: J Antimicrob Chemother. 2015 Nov 17;71(3):625–34. doi: 10.1093/jac/dkv376 (PMC4743696; doi:10.1093/jac/dkv376)
Supplement: Supplementary Data [file supp_dkv376_dkv376supp_Data1.docx]

**Supplementary data**

**Southern analysis of genomic DNA from nitro-drug-resistant and sensitive lines**

To investigate whether the *T. brucei* homologue of *TcNTR* has been affected in our NfxR and FxR cell lines in the process of generating such resistance, genomic DNA extracted from these cells, as well as from wild-type *T. brucei*, was analysed by Southern hybridisation using two reference genes (Figure S1). These genes were selected in order that one of them (*TbDHFR-TS*) was positioned on the same chromosome as *TbNTR* and the other (*TbTRYS*) was on a different chromosome. Furthermore, both *TbDHFR-TS* and *TbTryS* are single copy genes per haploid genome ^1-3^, and most importantly, they are thought to be unrelated to the mode of action and mode of activation of nitroaromatic pro-drugs. Following hybridisation with the labelled ORF of *TbNTR*, a band at the expected size of 6.8 kb was observed in all samples (Figure S1), which confirmed that at least one allele of *NTR* was present in both NfxR and FxR cell lines. Based on a visual comparison of the band corresponding to *TbNTR* and the bands corresponding to the two reference genes ratios between the resistant lines and wild-type *T. brucei* (Figure S1), it was evident that the *NTR* copy number in NfR1 and 2 cells had been reduced suggesting that these nitro drug resistant cell lines may have lost a single copy of the gene. However, in the FxR cell lines were found to maintain proportionally similar levels of all three genes as seen in WT cells, which would suggest that no change in the *NTR* copy number had occurred*.*

**
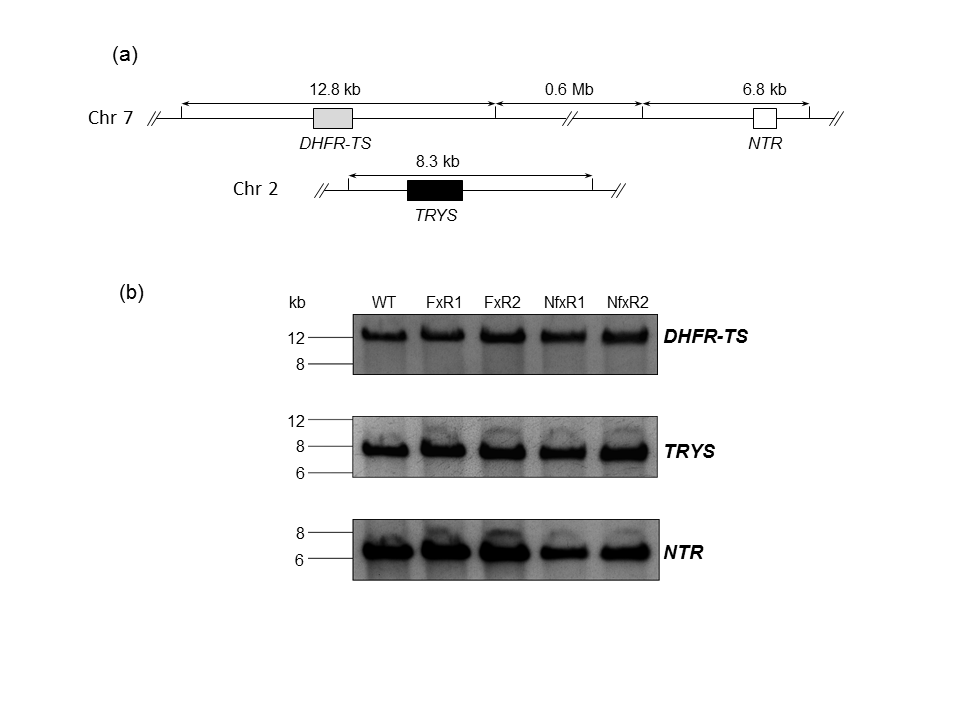
**

**Figure S1.** Analysis of NTR copy number in wild-type and resistant *T. brucei* lines. (A) Restriction map depicting the position of EcoRV sites (vertical lines) on chromosome 7 (top) and chromosome 2 (bottom) in *T. brucei* S427. Boxes represent the positions of the ORFs of TbDHFR-TS (Tb927.7.5480), TbNTR (Tb927.7.7230) and TbTRYS (Tb927.2.4370). (B) Southern analysis of wild-type and nitro drug resistant bloodstream trypanosomes. The membrane was probed with the DIG-labelled ORFs of TbDHFR-TS (top), TbTRYS (middle) and TbNTR (bottom).

**Table S1.** Cloning primers

Upper case letters refer to nucleotides corresponding to gene sequences in *T. brucei*; lower case refers to additional sequences used in generating constructs. Restriction endonuclease sites are underlined.

| **Primers** | **Sequence** |
| --- | --- |
| **NTR^SKO^** |  |
| NotI_5′UTR_F | 5′-ataagaatgcggccgcGAGGAAGGGACAACAGTGTACAGGG-3′ |
| 5′UTR_HindIII/PmeI_R | 5′-gtttaaacttacggaccgtcaagcttCTCGAGAGGGGACAAAGCCTCTCTC-3′ |
| PmeI/BamHI_3′UTR_F | 5′-gacggtccgtaagtttaaacggatccTGGTTGGATCTTGCAAAGGGAAAAGC-3′ |
| 3′UTR_NotI_R | 5′-ataagtaagcggccgcCGTACAGGAGAGTAGGTAGTATGTG-3′ |
| 3′UTR_mut_G89C_F | 5′-CTGACTGAGTGCCGCCGCAAAGGCG-3′ |
| 3′UTR_mut_G89C_R | 5′-CGCCTTTGCGGCGGCACTCAGTCAG-3′ |
| 5′UTR_-59_F | 5′-GGCGTTTATGAAGAATGTAAATG-3′ |
| 3′UTR_+600_R | 5′-CTCGCTCACTTCCTACTTCTG-3′ |
| 5′UTR_-107_F | 5′-GCTCTTGCTGCTTTGTACGCTC-3′ |
| 3′UTR_+152_R | 5′-CTGTACTGATGCATGTATTAC-3′ |
| **NTR^MUT^** |  |
| NTR^MUT^_F | 5′-CACGTCCCATCGACCACACT-3′ |
| NTR^MUT_^R | 5′-AGTGTGGTCGATGGGACGTG-3′ |
| **NTR^OE^** |  |
| NTR-HindIII-F | 5′- aagcttATGAACGTGAGCCGCTGCCG-3′ |
| NTR-PacI | 5′- ttaattaaTCAGAAGCGATTCCATCGGACG-3′ |
| **Tb927.7.7410^OE^** |  |
| 7410-HindIII_F | 5′-aagcttATGTCGAGGCTGTCTTTCGAAGGCGTG-3′ |
| 7410-PacI_R | 5′-ttaattaaCTTCTTGACAACAACAATACCCTTTG-3′ |
| **Tb927.1.1050^SKO^** |  |
| NotI_5′UTR | 5′-ataagaatgcggccgcACCATACCCTTTCGCATGTCGTACTT-3′ |
| 5′UTR_HindIII/PmeI_R | 5′-gtttaaacttacggaccgtcaagcttAAGCTTCCTTCAGTTATATGCTATCG-3′ |
| PmeI/BamHI_3′UTR_F | 5′-gacggtccgtaagtttaaacggatccTTAGGTAAAACAAAGGTCCCCATCC-3′ |
| 3′UTR_NotI_R | 5′-ataagtaagcggccgcGATGGTGAGCAGAGAAAGTGACTTGAG-3′ |
| **Tb927.7.7410^SKO^** |  |
| 5′UTR_-500_F  HYG R | 5′-CTTCACTTTGAATAGTCCAATCT-3′  5′-CTATTCTTTGCCCTCG-3′ |
| **qPCR** |  |
| TbNTR_F | 5′- TGCTGGATTGGATACGGCAA-3′ |
| TbNTR_R | 5′- GTTTCCCCTGCTCATCTGCT-3′ |
| TbTERT_F | 5′- GAGCGTGTGACTTCCGAAGG-3′ |
| TbTERT_R | 5′- AGGAACTGTCACGGAGTTTGC-3′ |

**Table S2.** Gene deletions on chromosome 7

| NfxR | FxR | GeneID | Gene Description |
| --- | --- | --- | --- |
| 2 copies | 2 copies | Tb927.7.7180 | leucine-rich repeat protein (LRRP), putative, leucine-rich repeat protein 1 (LRRP1), putative |
| gene copy lost (partially) | 2 copies | Tb927.7.7190 | hypothetical protein, conserved |
| gene copy lost | 2 copies | Tb927.7.7200 | hypothetical protein, conserved |
| gene copy lost | 2 copies | Tb927.7.7210 | hypothetical protein, conserved |
| gene copy lost | 2 copies | Tb927.7.7220 | hypothetical protein, conserved |
| gene copy lost | 2 copies | Tb927.7.7230 | nitroreductase, NADH dehydrogenase, putative (NTR) |
| gene copy lost | 2 copies | Tb927.7.7240 | leucine-rich repeat protein (LRRP), putative |
| gene copy lost | 2 copies | Tb927.7.7250 | hypothetical protein, conserved |
| gene copy lost | 2 copies | Tb927.7.7260 | kinesin, putative (TbKIF9B) |
| gene copy lost | 2 copies | Tb927.7.7270 | hypothetical protein, conserved |
| gene copy lost | 2 copies | Tb927.7.7280 | RNA-binding protein, putative |
| gene copy lost | gene copy lost (partially) | Tb927.7.7290 | hypothetical protein, conserved |
| gene copy lost | gene copy lost | Tb927.7.7300 | ATP-dependent DEAD/H RNA helicase, putative |
| gene copy lost | gene copy lost | Tb927.7.7310 | hypothetical protein |
| gene copy lost | gene copy lost | Tb927.7.7320 | hypothetical protein, conserved |
| gene copy lost | gene copy lost | Tb927.7.7330 | hypothetical protein, conserved |
| gene copy lost | gene copy lost | Tb927.7.7340 | hypothetical protein, conserved |
| gene copy lost | gene copy lost | Tb927.7.7350 | hypothetical protein |
| gene copy lost | gene copy lost | Tb927.7.7360 | cdc2-related kinase 2,cell division control protein 2 homolog 2 (CRK2) |
| gene copy lost | gene copy lost | Tb927.7.7370 | unspecified product |
| gene copy lost | gene copy lost | Tb927.7.7380 | U6 snRNA-associated Sm-like protein LSm3p (TbLSm3) |
| gene copy lost | gene copy lost | Tb927.7.7390 | hypothetical protein |
| gene copy lost | gene copy lost | Tb927.7.7400 | hypothetical protein, conserved |
| gene copy lost | gene copy lost | Tb927.7.7410 | oxidoreductase, putative |
| gene copy lost | gene copy lost | Tb927.7.7420 | ATP synthase alpha chain, mitochondrial precursor, ATP synthase F1, alpha subunit |
| gene copy lost | gene copy lost | Tb927.7.7430 | ATP synthase alpha chain, mitochondrial precursor, ATP synthase F1, alpha subunit |
| gene copy lost | gene copy lost | Tb927.7.7440 | hypothetical protein, conserved |
| gene copy lost | gene copy lost | Tb927.7.7450 | GTP-binding protein, putative |
| gene copy lost | gene copy lost | Tb927.7.7460 | hypothetical protein, conserved |
| gene copy lost | gene copy lost | Tb927.7.7470 | receptor-type adenylate cyclase GRESAG 4, putative |
| gene copy lost | gene copy lost | Tb927.7.7480 | trans-sialidase, putative |
| gene copy lost | gene copy lost | Tb927.7.7490 | hypothetical protein, conserved |
| gene copy lost | gene copy lost | Tb927.7.7500 | thymine-7-hydroxylase, putative (TLP7) |
| ND | ND | Tb927.7.7510 | hypothetical protein |
| ND | ND | Tb927.7.7520 | receptor-type adenylate cyclase GRESAG 4, putative |
| ND | ND | Tb927.7.7530 | receptor-type adenylate cyclase GRESAG 4, putative |
| ND | ND | Tb927.7.7540 | leucine-rich repeat protein (LRRP, pseudogene), putative, leucine-rich repeat protein 1 (LRRP1), degenerate |
| ND | ND | Tb927.7.7550 | hypothetical protein |
| ND | ND | Tb927.7.7560 | hypothetical protein |

**Table S3.** Genomic variants predicted to cause non-synonymous amino acid changes in NfxR and FxR proteins in comparison to WT (see Excel file). High confidence SNPs were defined as SNPs at positions for which a genotype was called for at least 2 of the 6 isolates plus the reference sample (WT). Also, no genotype disagreement was permitted within the 3 FxR isolates and within the 3 NfxR isolates, respectively.

**Table S4.** Genes with high-confidence SNPs in NfxR and FxR clones and their overlap with genes identified in a RITseq analysis ^4^ of nifurtimox

| **Gene** | **High-Confidence SNP(s) in NfxR** | **High-Confidence SNP(s) in FxR** | **In Horn Nfx  RIT-SEQ** | **Gene Description** |
| --- | --- | --- | --- | --- |
| Tb927.2.3270 | YES | - | - | 65 kDa invariant surface glycoprotein |
| Tb927.2.6320 | - | - | - | adenosine transporter 2, putative |
| Tb927.7.7300 | YES | YES | - | ATP-dependent DEAD/H RNA helicase, putative |
| Tb09.244.2410 | YES | YES | - | BARP protein |
| Tb927.10.6050 | YES | YES | - | clathrin heavy chain |
| Tb11.12.0001 | YES | YES | - | DNA polymerase kappa, putative |
| Tb11.01.6240 | YES | YES | - | expression site-associated gene (ESAG) protein, putative; expression site-associated gene 2 (ESAG2) protein, putative |
| Tb927.10.16190 | YES | YES | - | expression site-associated gene 4 (ESAG4) protein, putative; expression site-associated gene (ESAG) protein, putative; receptor-type adenylate cyclase |
| Tb927.7.7450 | YES | YES | - | GTP-binding protein, putative |
| Tb04.3I12.100 | YES | YES | - | hypothetical protein |
| Tb927.7.6580 | YES | - | - | hypothetical protein |
| Tb927.7.7310 | YES | YES | - | hypothetical protein |
| Tb927.7.7510 | YES | YES | - | hypothetical protein |
| Tb927.1.1650 | - | YES | - | hypothetical protein, conserved |
| Tb927.1.2990 | YES | YES | - | hypothetical protein, conserved |
| Tb927.1.530 | YES | - | - | hypothetical protein, conserved |
| Tb927.2.1460 | - | - | - | hypothetical protein, conserved |
| Tb927.4.5380 | YES | - | - | hypothetical protein, conserved |
| Tb927.5.1940 | YES | - | - | hypothetical protein, conserved |
| Tb927.6.2760 | YES | - | - | hypothetical protein, conserved |
| Tb927.6.620 | YES | - | - | hypothetical protein, conserved |
| Tb927.7.2410 | YES | YES | - | hypothetical protein, conserved |
| Tb927.7.7200 | YES | - | - | hypothetical protein, conserved |
| Tb927.7.7220 | YES | - | - | hypothetical protein, conserved |
| Tb927.7.7250 | YES | - | - | hypothetical protein, conserved |
| Tb927.7.7270 | YES | - | - | hypothetical protein, conserved |
| Tb927.7.7290 | YES | - | - | hypothetical protein, conserved |
| Tb927.7.7320 | - | - | - | hypothetical protein, conserved |
| Tb927.7.7330 | YES | YES | - | hypothetical protein, conserved |
| Tb927.7.7340 | YES | YES | - | hypothetical protein, conserved |
| Tb927.7.7400 | YES | YES | - | hypothetical protein, conserved |
| Tb927.7.7490 | YES | YES | - | hypothetical protein, conserved |
| Tb927.8.2220 | - | YES | - | hypothetical protein, conserved |
| Tb927.8.7490 | - | YES | - | hypothetical protein, conserved |
| Tb09.160.0750 | YES | YES | - | hypothetical protein, conserved |
| Tb09.160.1370 | YES | - | - | hypothetical protein, conserved |
| Tb09.211.3890 | - | YES | - | hypothetical protein, conserved |
| Tb927.10.12160 | - | YES | - | hypothetical protein, conserved |
| Tb927.10.13690 | YES | - | - | hypothetical protein, conserved |
| Tb927.10.15750 | - | - | - | hypothetical protein, conserved |
| Tb927.10.530 | - | - | - | hypothetical protein, conserved |
| Tb11.01.5240 | YES | - | - | hypothetical protein, conserved |
| Tb11.01.6870 | - | YES | - | hypothetical protein, conserved |
| Tb11.02.2220 | - | YES | - | hypothetical protein, conserved |
| Tb11.02.3060 | - | - | - | hypothetical protein, conserved |
| Tb11.52.0006 | - | - | - | hypothetical protein, conserved (pseudogene) |
| Tb927.8.1400 | YES | YES | - | hypothetical protein, conserved (pseudogene); hypothetical protein, conserved, degenerate |
| Tb927.7.7240 | YES | - | - | hypothetical protein, conserved; leucine-rich repeat protein (LRRP), putative |
| Tb09.160.5540 | - | YES | - | hypothetical protein, unlikely |
| Tb09.211.1560 | YES | YES | - | hypothetical protein, unlikely |
| Tb09.211.4050 | YES | YES | - | hypothetical protein, unlikely |
| Tb09.v2.0010 | YES | YES | - | invariant surface glycoprotein 100 (pseudogene), putative; invariant surface glycoprotein 100, point mutation |
| Tb927.7.7260 | YES | - | - | kinesin, putative |
| Tb927.8.6830 | - | - | - | kinesin, putative |
| Tb09.211.4960 | - | - | - | leucine-rich repeat protein (LRRP, pseudogene), putative; leucine-rich repeat protein (LRRP), point mutation |
| Tb927.3.3870 | YES | - | - | lipase domain protein, putative |
| Tb09.211.2900 | YES | YES | - | membrane transporter protein, putative |
| Tb927.8.5810 | YES | - | - | mitochondrial carrier protein, putative; mitochondrial carnitine/acylcarnitine carrier protein |
| Tb11.01.3750 | YES | - | - | molybdenum cofactor biosynthesis protein, putative |
| Tb11.02.5610 | - | YES | - | MSP-A, putative |
| **Tb927.7.7230** | **YES** | **-** | **YES** | **nitroreductase; NADH dehydrogenase, putative** |
| Tb927.7.7410 | YES | YES | - | oxidoreductase, putative |
| Tb927.10.12510 | YES | YES | - | P-type H+-ATPase, putative |
| Tb927.6.2250 | YES | - | - | rac serine-threonine kinase, putative; protein kinase, putative |
| Tb927.5.2850 | YES | YES | - | radial spoke protein RSP2, putative |
| Tb927.8.7910 | - | YES | - | receptor-type adenylate cyclase GRESAG 4 (pseudogene), putative; receptor-type adenylate cyclase GRESAG 4, frameshift |
| Tb927.6.270 | YES | - | - | receptor-type adenylate cyclase GRESAG 4, pseudogene, putative; receptor-type adenylate cyclase GRESAG 4, degenerate |
| Tb927.6.290 | - | - | - | receptor-type adenylate cyclase GRESAG 4, pseudogene, putative; receptor-type adenylate cyclase GRESAG 4, degenerate |
| Tb927.4.3880 | YES | YES | - | receptor-type adenylate cyclase GRESAG 4, putative |
| Tb927.6.300 | YES | - | - | receptor-type adenylate cyclase GRESAG 4, putative |
| Tb927.7.6050 | YES | YES | - | receptor-type adenylate cyclase GRESAG 4, putative |
| Tb927.7.7470 | YES | YES | - | receptor-type adenylate cyclase GRESAG 4, putative |
| Tb11.02.2380 | - | - | - | retrotransposon hot spot protein (RHS, pseudogene), putative; retrotransposon hot spot protein (RHS), degenerate |
| Tb927.7.1990 | - | YES | - | retrotransposon hot spot protein (RHS, pseudogene), putative; retrotransposon hot spot protein 7 (RHS7), point mutation |
| Tb927.4.5170 | YES | - | - | ribosomal protein L7/L12, putative |
| Tb927.7.7280 | YES | - | - | RNA-binding protein, putative |
| Tb927.10.9720 | YES | YES | - | RNA-editing-associated protein 1; RNA-binding protein; oligo (U) binding protein; RNA editing complex protein |
| Tb927.8.6930 | - | - | - | serine/threonine-protein kinase NrkA |
| Tb927.10.4180 | YES | - | - | TFIIF-stimulated CTD phosphatase, putative |
| Tb927.7.7500 | YES | YES | - | thymine-7-hydroxylase, putative |
| Tb927.7.7480 | YES | YES | - | trans-sialidase, putative |
| Tb927.7.6540 | YES | YES | - | variant surface glycoprotein (VSG, atypical), putative |

**Supplementary references**

1. Gamarro F, Yu PL, Zhao J, et al. *Trypanosoma brucei* dihydrofolate reductase-thymidylate synthase: Gene isolation and expression and characterization of the enzyme. *Mol Biochem Parasitol* 1995; **72**: 11-22.

2. Gamarro F, Yu PL, Zhao J, et al. Erratum: *Trypanosoma brucei* dihydrofolate reductase-thymidylate synthase: Gene isolation and expression and characterization of the enzyme (Molecular and Biochemical Parasitology (1995) Vol. 72 (pp. 11-22)). *Mol Biochem Parasitol* 1995; **75**: 127.

3. Oza SL, Ariyanayagam MR, Aitcheson N, et al. Properties of trypanothione synthetase from *Trypanosoma brucei*. *Mol Biochem Parasitol* 2003; **131**: 25-33.

4. Alsford S, Eckert S, Baker N, et al. High-throughput decoding of antitrypanosomal drug efficacy and resistance. *Nature* 2012; **482**: 232-6.
